# Effect of high hydrostatic pressure on aroma volatile compounds and aroma precursors of Hami melon juice

**DOI:** 10.3389/fnut.2023.1285590

**Published:** 2023-11-10

**Authors:** Longying Pei, Wei Liu, Luxi Jiang, Heng Xu, Luping Liu, Xiaoyu Wang, Manli Liu, Buhailiqiemu Abudureheman, Heng Zhang, Jiluan Chen

**Affiliations:** ^1^College of Food Science and Engineering, Xinjiang Institute of Technology, Aksu, Xinjiang Province, China; ^2^College of Food Science and Engineering, Tarim University, Alar, Xinjiang Province, China; ^3^School of Food Science and Technology, Shihezi University, Shihezi, Xinjiang Province, China

**Keywords:** Hami melon juice, high hydrostatic pressure, aroma enzymes, aroma volatile compounds, amino acids, fatty acids

## Abstract

High hydrostatic pressure (HHP) treatment is an effective technique for processing heat-sensitive fruits and causes changes in volatile compounds and their precursors while maintaining quality. We investigated the changes and correlations of volatile compounds, related enzyme activities and precursor amino acids, and fatty acids in Hami melon juice under 350–500 MPa pressure. The application of HHP treatment resulted in a considerable reduction of esters and a substantial increase in aldehydes and alcohols in C6 and C9. Activities of lipoxygenase (*LOX*), alcohol acyltransferase (*AAT*), and phospholipase A2 (*PLA2*) were lower than those of the untreated group, alcohol dehydrogenase (*ADH*) activity was reversed. When compared to fresh cantaloupe juice, there was an increase in both the types and contents of amino acids with lower total fatty acid contents than the control group. Positive correlations were observed among six ester-related substances and eight alcohol-related substances. Additionally, the correlations between volatile compounds and fatty acids were more substantial compared to those between volatile compounds and amino acids. HHP treatment increases Hami melon flavor precursors and is an effective way to maintain the aroma volatile compounds and flavor of Hami melon juice.

## 1. Introduction

Hami melon (*Cucumis melo* L. var. reticulatus naud.) is a characteristic fruit of Xinjiang Uygur Autonomous Region, a melon with a thick skin, oval shape, reticulated rind, sweet taste and pleasant aroma. In addition, the unique geographical environment facilitates photosynthesis of the fruit during the day, reduces respiratory consumption, and deposits more nutrients at night, such as long daylight hours and a DIF (diurnal temperature difference) of about 15°C, which is particularly suitable for cultivation and development of Hami melon (1). As a special agricultural product of Xinjiang, the annual production of this melon is about 2.34 million tons, but it is rather intolerant to storage and transportation. In addition, it is a heat-sensitive fruit that causes severe flavor losses during thermal processing, such as a 20% reduction in total esters and a large reduction in the content of C6 and C9 alcohols and aldehydes after heat treatment (2), so it is important to find an innovative food process to preserve its nutritional and unique flavor.

High hydrostatic pressure (HHP) is a non-thermal processing technology that uses water as a medium to treat raw materials under ultrahigh pressure (100–1,000 MPa) at room temperature or under mild conditions, raw materials treated in this way can effectively avoid the loss of nutrients and retain their freshness, flavor and color (1). HHP can be able to destroy cellular tissue, inactivate microorganisms, in fruit and vegetable processing is mainly used to sterilize (3), improve the extraction rate of bioactive substances (4) and passivation of enzyme activity (5). Furthermore, HHP treatment can be carried out at room temperature, which reduces the heating and cooling of the required energy consumption, and not in direct contact with the processing equipment, avoiding the occurrence of secondary contamination after pasteurization, and can achieve the recycling of pressure transfer media, it also has the advantages of lower energy consumption and pollution, is a relatively environmentally friendly processing technology (6). Mild heating is another way to improve the efficiency of high pressure enzyme inactivation, study have shown that a better degree of microbial and enzyme inactivation can be achieved at lower pressures (300–500 MPa) combined with mild heating (40–70°C) treatment. Moreover, the retention and activation of enzymes by high pressure also contribute to improving the texture of processed fruit and vegetable products, creating new structures that are different from those achieved by thermal processing (7), this was demonstrated in our previous study, in which Hami melon juice treated at 45°C significantly controlled the level of microbial and some enzyme activities by combining HHP treatment with different temperatures (25–55°C), while maintaining the original good quality (8). The changes in aroma components and catalytic reactions of flavor enzymes when fruit and vegetable juices are treated under ultrahigh pressure inevitably have their characteristics and patterns (9, 10). On the one hand, the form and structure of aroma components in fruit and vegetable juices treated by ultrahigh pressure underwent different degrees of change (11, 12). On the other hand, flavor enzymes in fruit and vegetable juices treated with HHP are partially inactivated or activated, further influencing the composition and content of the catalyst (13). The effect of residual enzyme activity on the actual fruit system during or after HHP may be a contributing factor (14).

Some compounds of the aliphatic series or aromatic groups, including esters, alcohols, acids, and aldehydes, are products or intermediates in the metabolism of amino acids, are usually found in animals and plants in the form of protein structures, bound to other macromolecules or free amino acids. Differences in food system processing and storage conditions, such as temperature, time, pressure, and substrate, will result in different changes in amino acid and protein structures (15). Fatty acids are precursors of many flavor substances and play a key role in the flavor changes of fruits. Fatty acids mainly produce some branched chain fatty alcohols, aldehydes, ketones and esters during their metabolism. The fresh odor in fruits and vegetables comes from C6 and C9 aldehydes and alcohols, which are converted from fatty acids to hydroperoxides by *LOX* and then converted to the corresponding aldehydes and alcohols by oxidation, dissociation and dehydrogenation to form esters, among others (16).

To assess the alterations in the aroma substances of Hami juice following HHP treatment, we researched the correlation between the juice’s aroma compounds, precursors (fatty acids and amino acids), and associated changes in enzyme activity. Four different pressures were employed to treat Hami melon juice. The study analyzed the changes in aroma precursors, the enzymes involved in relevant metabolic pathways and the volatile compounds. Furthermore, the correlation between the four substances throughout the metabolic process was explored using correlation analysis. This provides a theoretical basis for the effect of HHP treatment on the flavor substances of Hami melon juice.

## 2. Materials and methods

### 2.1. Preparations of melon samples and melon juice

Nine boxes of Hami melons were selected at random from “Fruit and Vegetable Wholesale Market” in Shihezi City. These late-maturing melons were grown in Jiashi County, Xinjiang province, and were fully mature, with no visible lesions or rot. The soluble solid content of melons was approximately 12% with a pH range of 5.6 to 5.8. The melons were then placed in pre-sterilized cold storage at 4°C, to keep them fresh for later use. Hami melon juice was processed using a HHP machine (HHP.L.2–600/0.6, Huatai Senmiao Biotechnology Co., Tianjin, China).

### 2.2. High hydrostatic pressure treatment

Using water as the pressure transmission medium, four pressure gradients were set, 350, 400, 450 and 500 MPa, the temperature and pressure were kept at 45°C and 15 min, and the sub-packed Hami melon juice was put into the autoclave after the temperature of the ultrahigh pressure sterilizer was raised to 45°C by hot water circulation, then its water level was adjusted, and the pressure was set for 15 min, then the pressurization started, and after completion release the pressure automatically. Put the processed samples into the refrigerator at 4°C and stand for no more than 4 h from placement to measurement.

### 2.3. Extraction of volatiles compounds and analysis of GC–MS

After HHP processing, volatiles were extracted from juice by HS-SPME, by using SPME (Supelco, Inc.) after preprocessing polydimethylsiloxane (PDMS) fiber at 250°C for 30 min. Eight mL juice was transferred quickly to a headspace bottle (15 mL) containing 2.1 g NaCl, the juice was equilibrated through magnetic heating plate (HMS-901D, Bdjk Biotechnology Co., Ltd.) at 40°C for 10 min. The volatile components in the headspace were extracted at 40°C for 30 min by a stable flex PDMS fiber placed at 1 cm away from the liquid surface under 100 rpm/min magnetic stirring.

GC–MS analysis was performed by GC–MS (HP 7890/5975, Agilent Technologies) equipped with DB-5 (5% cross-linked phenylmethyl silicone) column (30 m × 0.25 mm i.d. × 0.25 μm, Agilent Technologies). The fiber was inserted into the sample hole and desorbed at 250°C for 1 min and run for 30 min. The fiber head is held for 5 min to remove impurities in the sample hole, injected in splitless mode. The carrier gas (He, 99.999%) is added for 1 min and the flow rate was set to 40 cm/s. The initial temperature of the GC is maintained at 50°C for 1 min, then raised to 100°C (5°C/min), then raised to 250°C (10°C/min) and maintained for 9 min. The carrier gas was Helium (1.2 mL/min) with splitless injection. The quadrupole mass spectrometer was utilized in 70 eV electron ionization mode, with an ion source temperature of 200°C, a quadrupole temperature of 106°C, and a continuous scan range of 33–350 m/z.

### 2.4. Determining method of *ADH* activity

The *ADH* crude enzyme extract was extracted with a modification of the method described by Ntsoane et al. (17), combing Hami melon juice (4 mL) and 12 mL *ADH* extraction solution containing 10 mL of MES buffer (100 mM, pH = 6.5), 2 mM DTT, 1% (w/v) PVPP in a centrifuge tube, then stirred well to digestion (4°C, 30 min). The solution was centrifuged (GR21G, Hitachi Koki Co., Ltd., Tokyo, Japan) for 30 min (12,000 × g, 4°C), the supernatant as crude enzyme extract stored at 4°C for use. Two-point five mL MES buffer (100 mM pH = 6.5), 0.15 mL NADH (1.6 mM), 0.15 mL acetaldehyde (80 mM) and finally 0.3 mL of crude enzyme extract were added and completely mixed. The *ADH* activity was determined by measuring the change in absorbance value at 340 nm (recorded every 20 s for 3 min).

### 2.5. Determination method of *LOX* activity

According to Zhang et al. (18) methodology with modifications. Briefly, The *LOX* assay utilized sodium linoleate as a substrate. To prepare, 60 μL linoleic acid (LA) is extracted and placed in nitrogen gas at 15°C, degassed distilled water (4 mL) was added to the LA together with Tween 20 (120 μL), then 60 μL NaOH (5 M) is added and the solution is diluted to 25 mL with distilled water. Next, a mixture of Hami melon juice (3 mL) and 3 mL of *LOX* extraction buffer [with 1% (*w/v*)TritonX-100, 1% (*w/v*) PVP, 0.05 M phosphate buffer at pH = 6.8] was combined and left for 30 min. The supernatant was stored at 4°C after subjecting the mixture to centrifugation (12,000 rpm/min, 4°C, 10 min). Next, enzyme extract (0.02 mL) was added into a quartz cuvette containing the reacting mixture, which consisted of sodium linoleate (0.1 mL) and 2.88 mL of borate buffer (0.05 M, pH = 6.8). The *LOX* activity was determined by measuring the change in absorbance value at 234 nm (recorded every 20 s for 3 min).

### 2.6. Determination method of *ATT* activity

The *AAT* crude enzyme extract was extracted and assayed by the Zhu et al. (19) method with modifications. Hami melon juice (8 mL), potassium phosphate solution (16 mL, 100 mM, pH = 7.5) and 2.5 g PVPP were mixed thoroughly and digested (4°C, 30 min), then centrifuged for 30 min (12,000 × g, 4°C), the supernatant as crude enzyme extract stored at 4°C for use. Two point two 5 mL potassium phosphate buffer (100 mM, pH = 7.5), 0.3 mL DTNB (10 mM), 0.03 mL MgCl_2_ (1 M), 0.06 mL isoamyl alcohol (20 mM), 0.06 mL acetyl coenzyme (50 mM) and finally 0.3 mL of crude enzyme extract were mixed thoroughly. The *ATT* activity was determined by measuring the change in absorbance value at 234 nm (recorded every 20 s for 3 min).

### 2.7. Determination method of *PLA2* activity

Hami melon juice (3 mL) was homogenized in 5 mL of pre-cooled 0.1 M phosphate buffered saline (Na_2_HPO_4_·12H_2_O, NaH_2_PO4·2H_2_O). The mixture was centrifuged (12,000 × g, 4°C, 2 min). The supernatant was immediately collected as crude enzyme extract, and *PLA2* activity was detected by ELISA kit (DUMABIO, Shanghai, China). During the unit time, the absorbance variation 0.001 of 1 mL of enzyme solution was defined as 1 mL of *PLA2* activity.

### 2.8. Analysis of amino acids

Hami melon juice sample’s amino acid content was analyzed using an amino acid analyzer (L-8900, Hitachi) in accordance with the Chinese Standard (GB/T 5009.124–2016). Following hydrolysis into free amino acids using HCl, the proteins in the juice samples were derivatized with ninhydrin solution, separated on an ion-exchange column, and their spectrophotometric absorbance was measured at 440/570 nm. To determine the amino acid content (g/100 g), the retention time of each amino acid was measured and compared to the standard mixed solution.

### 2.9. Analysis of fatty acids

The fatty acids were determined using the third method outlined in the Chinese Standard (GB/T 5009.168–2016), the specific methodology is based on our previous research (8). For total lipid extraction, 50 mL of the juice was mixed with 10 mL of 95% ethanol. The resulting mixture was then transferred using a separatory funnel containing a 50 mL mixture of ethyl ether/petroleum ether and shaken for 5 min before being left to settle for 10 min. The extract from the ether layer was gathered in a 250 mL flask and dried using rotary evaporation, resulting in the fat extract. The fat extract was mixed with an 8 mL solution of 2% (*w/v*) NaOH in methanol, thereafter converting the total fatty acid into fatty acid methyl esters. The organic phase was dried using nitrogen stream, upon which 20 mL of n-heptane was added and shaken for 2 min, followed by the addition of saturated aqueous NaCl. After allowing the mixture to stand and stratify, 5 mL of the upper heptane extraction solution was transferred into a 25 mL test tube. Around 3–5 g of anhydrous sodium sulfate was added, shaken for 1 min, and left untouched for 5 min, before transferring the upper phase of the solution into the sample bottle.

Fatty acid analysis was performed using a Shimadzu GC-2010, which was equipped with a capillary column (100 m × 0.2 μm × 0.25 mm i.d). The detector and sample injector were set to temperatures of 280°C and 270°C, respectively. The heating protocol proceeded as follows: the initial temperature was set to 100°C and maintained for 13 min, then increased to 180°C (10°C/min), then held for 6 min. Subsequently, the temperature was further raised to 200°C (1°C/min) and held for a duration of 20 min. Finally, the temperature was increased to 230°C (4°C/min) and kept for a total of 10.5 min. The results of the analysis of fatty acids were presented in percentage content (percentage of certain fatty acids to total fat).

### 2.10. Statistical analysis

Data analysis utilized SPSS to analyze variance, Origin was adopted for charting. and all experiments were repeated three times. GC–MS Data was acquired using HP ChemStation software (Agilent Technologies) and compared to a NIST library to determine the composition. Spectral library was used initially to determine the composition, while retention times, mass spectra, actual compositions, and retention indices were used for determine most compositions, quantification of the components was achieved by adding n-heptanol as an internal standard at a certain concentration. Relative quantification was then performed using the area normalization method. Moreover, the principal component analysis used the ade4 package of R-3.5.3 language for drawing, and thermograph adopted the complex heatmap package of the R-3.5.3 language for charting. Correlation analysis made use of spearman for correlation analysis and cytoscape for visualization.^1^

## 3. Results

### 3.1. Change in aroma volatile compounds

As shown in Table 1, there were 30 compounds in the untreated group, mainly including 20 esters, 4 alcohols, 3 aldehydes and 3 ketones. There were 30, 31, 29 and 25 categories of compounds in the group from 350 to 500 MPa. The 350 MPa group contained 18 esters, 4 alcohols, 5 aldehydes, 3 ketones. The 400 MPa group contained 18 esters, 4 alcohols, 6 aldehydes, 3 ketones, the 450 MPa group contained 17 esters, 4 alcohols, 5 aldehydes, 3 ketones, the 500 MPa group contained 15 esters, 3 alcohols, 4 aldehydes, 3 ketones. Compared to the untreated group, at different pressures, the treated groups were reduced by 2 to 4 esters and their content decreased significantly. Additionally, the content of alcohols and aldehydes increased after treatment at different pressures, but the content of alcohols decreased with increasing pressure and the content of aldehydes increased significantly.

**Table 1:** Aroma volatile compounds in Hami melon juice under HHP treatments.

Volatile compound	Retention time (min)	Untreated	350 MPa	400 MPa	450 MPa	500 MPa
Eaters						
Methyl acetate	1.43	2.03 ± 0.57	ND	ND	ND	ND
Ethyl acetate	1.65	10.09 ± 0.43 ^a^	26.23 ± 0.99 ^b^	30.17 ± 1.5 ^c^	29.51 ± 0.91 ^c^	28.6 ± 1.11 ^c^
Propyl acetate	2.25	1.56 ± 0.42 ^a^	2.31 ± 0.19 ^bc^	3.09 ± 0.62 ^c^	2.97 ± 0.15 ^c^	2.76 ± 0.64 ^c^
Methyl butyrate	2.38	1.92 ± 0.49	ND	ND	ND	ND
Isobutyl acetate	2.67	3.91 ± 0.34 ^c^	1.80 ± 0.04 ^ab^	1.71 ± 0.02 ^a^	2.06 ± 0.12 ^b^	2.10 ± 0.16 ^b^
2-methyl propyl acetate	2.8	8.22 ± 1.87 ^c^	5.45 ± 0.38 ^b^	4.21 ± 1.07 ^b^	ND	4.91 ± 1.31 ^b^
Methyl 2-methylbutyrate	2.86	4.93 ± 0.87	ND	ND	ND	ND
Ethyl butanoate	3.25	5.69 ± 0.78 ^a^	5.98 ± 0.13 ^a^	5.84 ± 0.13 ^a^	6.8 ± 0.1 ^b^	5.79 ± 0.15 ^a^
Ethyl benzoate	3.45	1.08 ± 0.03 ^a^	0.98 ± 0.12 ^a^	0.96 ± 0.08 ^a^	0.98 ± 0.12 ^a^	0.98 ± 0.12 ^a^
Methyl valerate	3.64	0.09 ± 0.02	ND	ND	ND	ND
Ethyl 2-methylbutanoate	3.87	3.40 ± 0.57 ^a^	4.18 ± 0.23 ^b^	4.67 ± 0.46 ^bc^	4.36 ± 0.18 ^bc^	4.93 ± 0.12 ^c^
2-methyl butyl acetate	4.36	11.7 ± 2.55 ^b^	5.33 ± 0.31 ^a^	3.45 ± 0.65 ^a^	4.33 ± 0.56 ^a^	4.77 ± 1.24 ^a^
Methyl ethyl thioacetate	6.65	0.05 ± 0.01^a^	1.79 ± 0.15 ^b^	2.16 ± 0.38 ^b^	2.12 ± 0.29 ^b^	ND
Ethyl caproate	6.99	4.83 ± 0.78 ^b^	1.68 ± 0.21 ^a^	1.54 ± 0.44 ^a^	1.67 ± 0.23 ^a^	1.65 ± 0.42 ^a^
3-hexenol acetate	7.02	ND	1.16 ± 0.09 ^d^	0.66 ± 0.16 ^b^	0.86 ± 0.13 ^c^	0.1 ± 0.02 ^a^
Hexyl acetate	7.79	9.39 ± 0.83 ^c^	0.15 ± 0.03 ^a^	0.17 ± 0.15 ^a^	0.17 ± 0.03 ^a^	2.30 ± 0.49 ^b^
2,3-butanediol diacetate	8.68	0.29 ± 0.04 ^b^	1.16 ± 0.05 ^c^	0.20 ± 0.02 ^a^	0.24 ± 0.03 ^ab^	0.19 ± 0.03 ^a^
2-butanol-2 methyl acetate	8.83	ND	0.18 ± 0.03 ^c^	ND	0.11 ± 0.03 ^b^	0.1 ± 0.03 ^b^
Heptyl acetate	9.95	0.17 ± 0.03	ND	ND	ND	ND
Methyl phenylacetate	11.34	3.99 ± 0.16 ^a^	2.34 ± 0.44 ^c^	1.54 ± 0.03 ^b^	0.74 ± 0.57 ^a^	1.29 ± 0.01 ^ab^
Dimethyl 2-methylpropionate	15.4	ND	0.46 ± 0.04 ^c^	0.52 ± 0.08 ^c^	0.38 ± 0.03 ^b^	ND
Butyl butyrate	15.42	ND	1.18 ± 0.03 ^d^	0.56 ± 0.07 ^c^	0.44 ± 0.06 ^b^	0.4 ± 0.09 ^b^
Diethyl phthalate	18.64	0.15 ± 0.01 ^b^	0.13 ± 0.02 ^ab^	0.10 ± 0.03 ^a^	0.13 ± 0.03 ^ab^	0.24 ± 0.04 ^c^
Isopropyl palmitate	34.11	0.26 ± 0.05	ND	ND	ND	ND
Alcohols						
Ethanol	1.34	ND	6.40 ± 0.25 ^d^	3.29 ± 0.13 ^b^	4.36 ± 0.22 ^c^	ND
2-ethyl-1-hexanol	7.64	0.68 ± 0.13 ^a^	1.37 ± 0.08 ^b^	1.38 ± 0.03 ^b^	1.39 ± 0.06 ^b^	1.38 ± 0.07 ^b^
Nonanol	9.36	0.44 ± 0.07	ND	ND	ND	ND
(*Z*)-3-nonen-1-ol	11.5	ND	2.24 ± 0.12 ^b^	2.46 ± 0.03 ^c^	2.49 ± 0.07 ^c^	2.59 ± 0.06 ^c^
(*Z*)-6-nonen-1-ol	11.94	1.93 ± 0.10	ND	ND	ND	ND
2,7-octanediol	12.15	2.05 ± 0.15 ^a^	3.01 ± 0.07 ^b^	3.02 ± 0.08 ^b^	3.42 ± 0.08 ^c^	3.48 ± 0.06 ^c^
Aldehydes						
2,4-pentadienal	3.31	6.13 ± 0.22	ND	ND	ND	ND
Hexanal	4.93	ND	0.11 ± 0.06 ^b^	0.15 ± 0.03 ^b^	ND	ND
Heptanal	7.86	ND	0.24 ± 0.04 ^b^	0.30 ± 0.05 ^b^	0.44 ± 0.10 ^c^	ND
Nonanal	9.82	1.3 ± 0.06 ^d^	ND	0.14 ± 0.01 ^b^	0.22 ± 0.02 ^c^	0.14 ± 0.02 ^b^
(*E*)-6-nonenal	10.12	ND	7.52 ± 0.08 ^b^	9.39 ± 0.01 ^c^	10.51 ± 0.04 ^d^	11.6 ± 0.35 ^e^
Benzaldehyde	10.23	ND	0.12 ± 0.01 ^b^	0.22 ± 0.03 ^d^	0.15 ± 0.01 ^b^	0.16 ± 0.01 ^b^
(2*E*,6*Z*)-nonadienal	11.65	ND	3.15 ± 0.07 ^b^	3.15 ± 0.15 ^b^	4.55 ± 0.11 ^c^	3.15 ± 0.02 ^b^
Ketones						
6-methyl-5-heptene-2-one	7.34	0.16 ± 0.01 ^a^	0.16 ± 0.01 ^a^	0.15 ± 0.01 ^a^	0.15 ± 0.02 ^a^	0.15 ± 0.01 ^a^
6,10-dimethylundeca-5,9-dien-2-one	16.96	0.25 ± 0.05 ^c^	0.20 ± 0.02 ^b^	0.19 ± 0.01 ^ab^	0.16 ± 0.01 ^ab^	0.15 ± 0.02 ^a^
2,2,6-trimethyl-3-butanedione	17.52	0.17 ± 0.03 ^c^	0.16 ± 0.02 ^c^	0.15 ± 0.01 ^bc^	0.12 ± 0.01 ^ab^	0.11 ± 0.02 ^a^

Results are presented in relative percentages and mean values ± standard deviation, *n* = 3. Volatile compound content is the relative percentage (%). ND, not detected. Different letters in the same raw correspond to significant differences (*p* < 0.05).

### 3.2. Changes in enzyme activity

**Figure 1:**
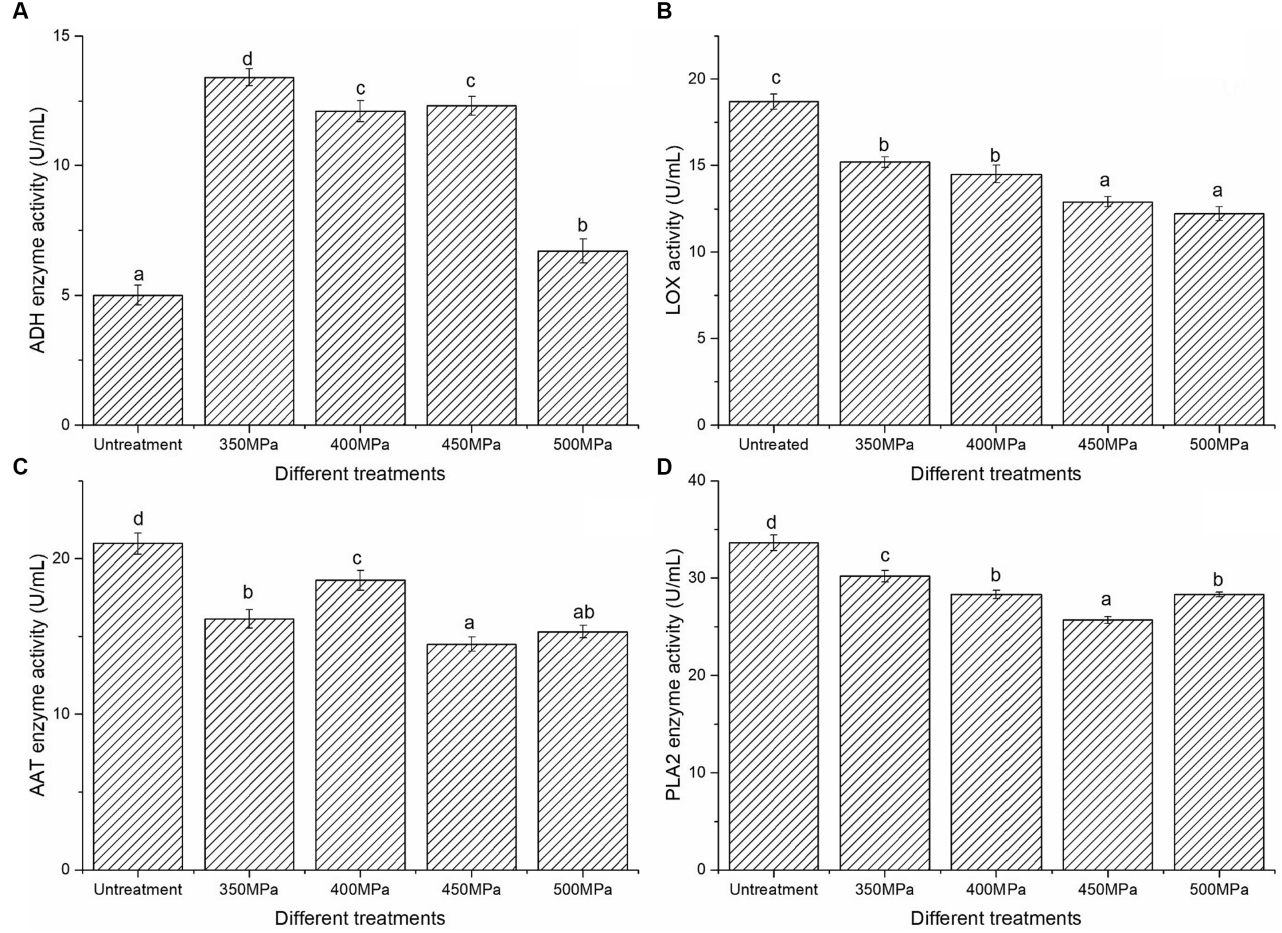
Effect of ADH **(A)**, LOX (B), AAT (C) and PLA2 (D).

The major enzymes generated by the metabolism of aromatic substances included *ADH*, *LOX*, *AAT* and *PLA2*. Compared with untreated group, all the activities of *ADH* (Figure 1A) increased significantly (*p* < 0.05) under HHP treatments, of which the activity was highest under 350 MPa (13.4 U/mL), the activity of *ADH* decreased with increasing pressure. The activity of *LOX* after HHP treatment was lower than that of the untreated group and decreased with increasing pressure (Figure 1B). The activity of *ADH* decreased in samples between 400 MPa and 450 MPa, while it decreased significantly in samples at 500 MPa (*p* < 0.05), with an activity of 6.7 U/mL. The activity of *AAT* after HHP treatment was much lower than in the untreated group. Furthermore, the activity of *AAT* (Figure 1C) was obviously different between 4 types of pressure (*p* < 0.05), of which the activity of *AAT* was highest in the 400 MPa (18.6 U/mL). In addition, the activity of *PLA2* (Figure 1D) was greatly reduced (*p* < 0.05), and it also showed a downward trend with increasing pressure, but got some upturn at 500 MPa.

### 3.3. Change in amino acid contents

**Figure 2:**
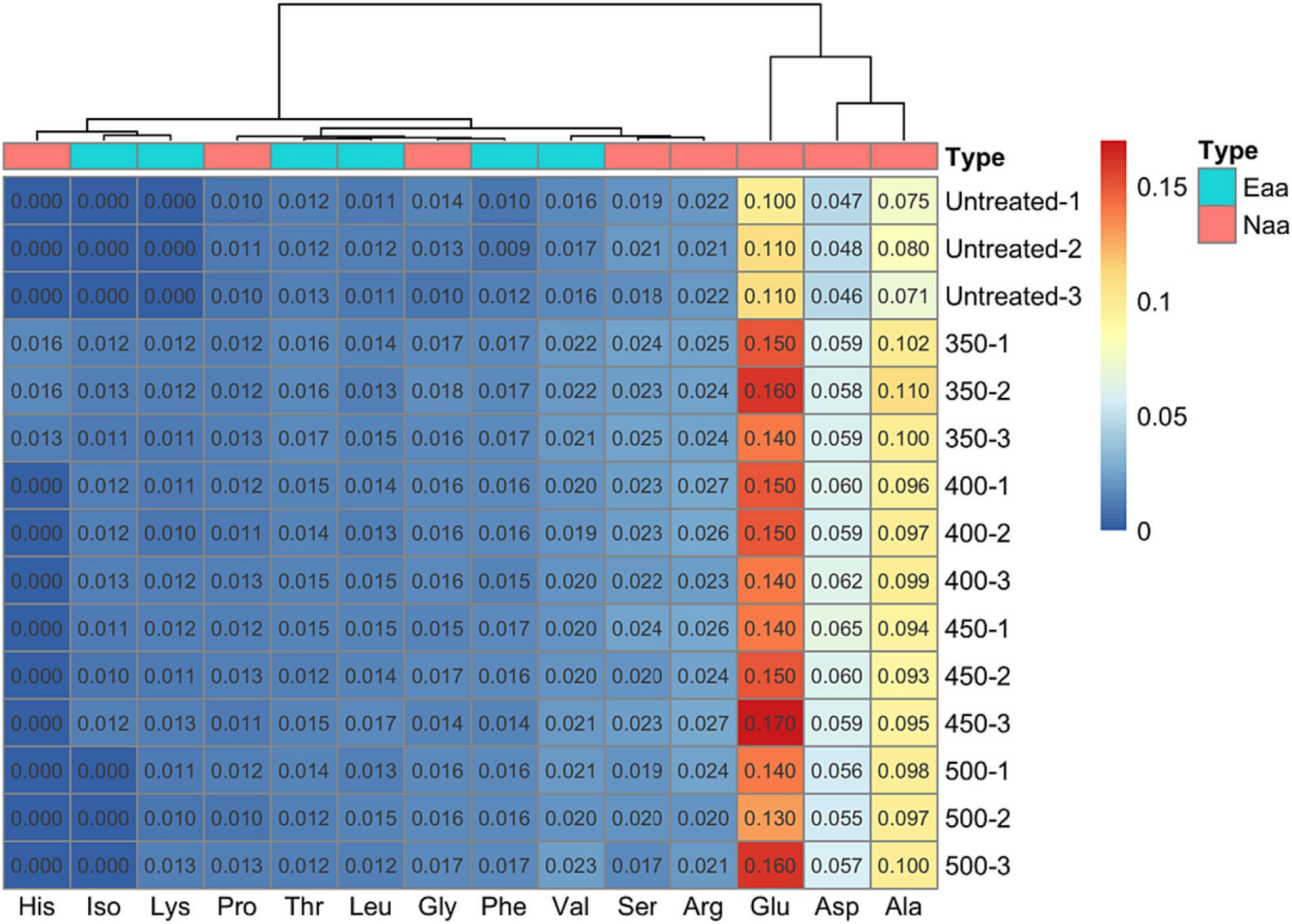
Changes of amino acids under different treatments.

As shown in Figure 2, untreated Hami melon juice is rich in 11 amino acids, namely aspartic acid (Asp), threonine (Thr), serine (Ser), glutamic acid (Gly), proline (Pro), glycine (Glu), alanine (Ala), valine (Val), leucine (Leu), phenylalanine (Phe) and arginine (Arg). Among these, there are 4 essential amino acids and 7 non-essential amino acids. The number of amino acids increases with increasing pressure. 350 MPa treatment contained 14 amino acids, with increases in histidine (His), isoleucine (Iso) and lysine (Lys) compared to the untreated group. 400 MPa and 450 MPa treatments contained 13 amino acids with increases in Iso and Lys. 500 MPa treatment contained 12 amino acids, with a higher level of Lys. Across all treatments, Glu, Asp and Ala had notably higher contents when compared to other amino acids. However, the concentrations of Pro, Glu, Lys and Phe remained unchanged as pressure increased. Essential and non-essential amino acids, as well as total amino acids, experienced a marked increase in HHP treated groups. Nevertheless, there was an observed tendency toward decreasing contents with greater pressure, with the 400 and 450 MPa treatment groups demonstrating a decreasing trend, no significant differences were found between the 400 and 450 MPa treatment groups.

### 3.4. Change in fatty acids contents

**Figure 3:**
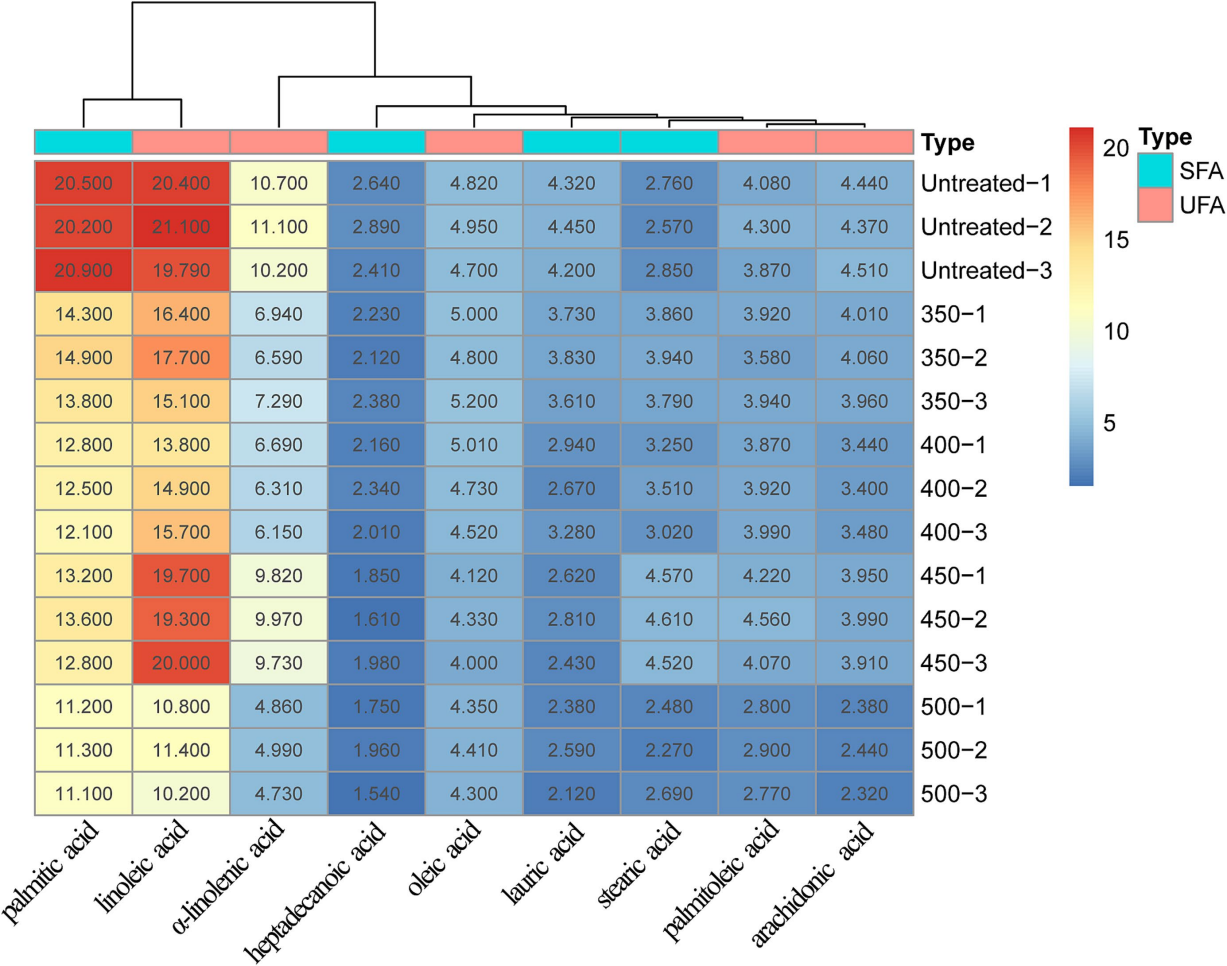
Changes of fatty acids under different treatments.

Hami melon juice was analyzed for its fatty acid composition and a total of nine fatty acids were identified, comprising five unsaturated and four saturated fatty acids (Figure 3). Within the untreated group, palmitic acid and linoleic acid had the highest fatty acid content, 20.53 and 20.33% respectively, and were significantly reduced after HHP treatment (*p* < 0.05), heptadecanoic acid had the lowest content (2.65%), accounting for only 3.55% of the total fatty acid content. In the HHP treatment group, linoleic acid had the highest quantity followed by palmitic acid and α-linolenic acid in underwent treatment at 450 MPa. When the pressure was increased from 350 MPa to 500 MPa, the levels of palmitoleic, linoleic acid and α-linolenic acid significantly rose, with the exception of 450 MPa. Conversely, the levels of other fatty acids declined with an increase in pressure.

### 3.5. Correlation analysis

**Figure 4:**
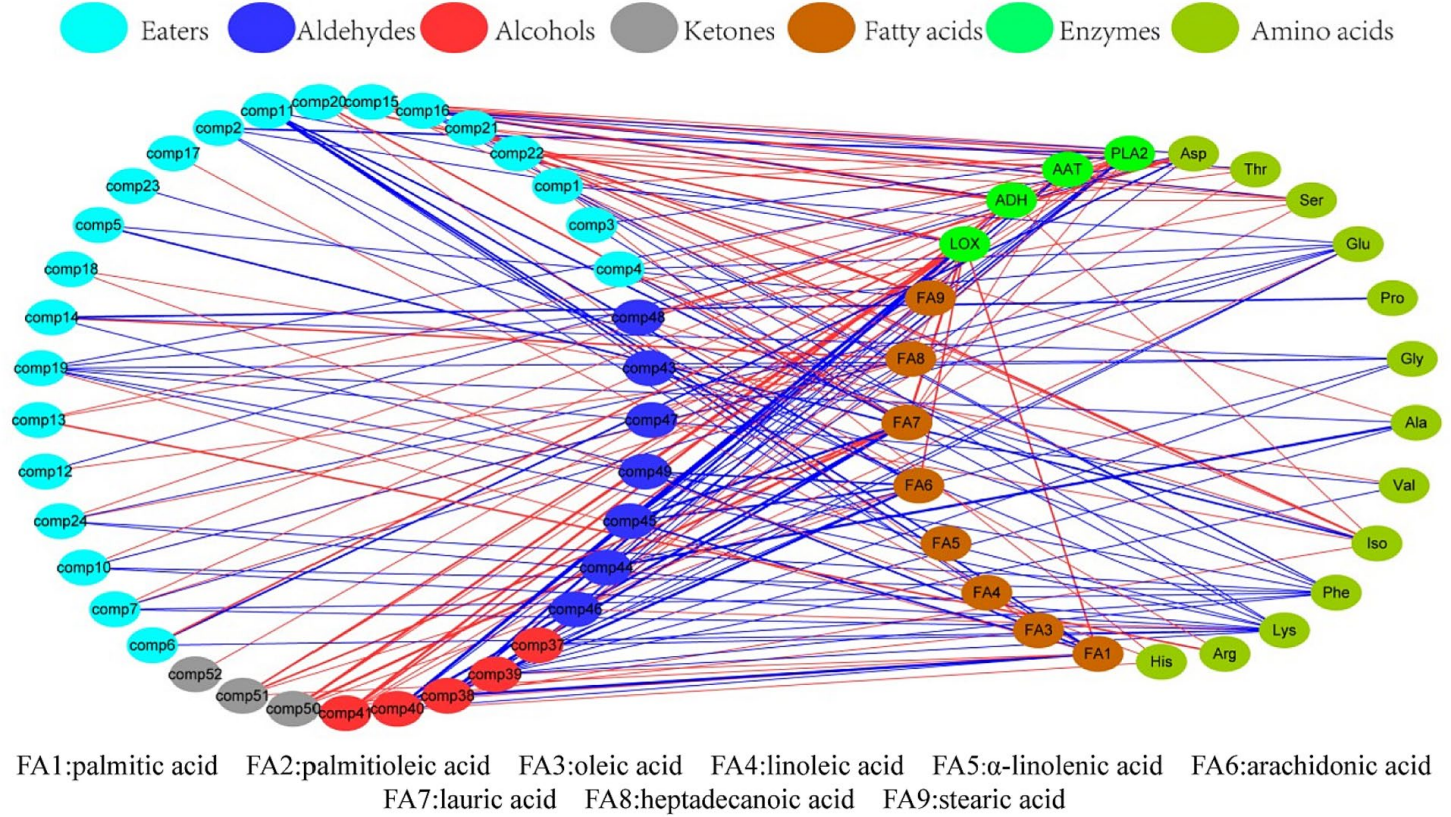
Correlation analysis of volatile compounds, amino acids, fatty acids and enzymes.

Figure 4 shows the correlation between aroma volatile compounds, amino acids, fatty acids and enzymes, with the red line indicating a positive correlation and the blue line a negative correlation. *PLA2* had the most related volatile compounds (nine esters, three alcohols, two aldehydes and two ketones), of which 10 were positively and 6 negatively correlated. Lys was associated with 12 volatile compounds, 11 of which were negatively correlated while only one exhibited a positive correlation, namely *2E*, *6Z*-nonadienal. Palmitic acid was found to have a correlation with 11 volatile compounds, of which six showed a negative correlation, while five showed a positive correlation. Based on the analysis in the right circle, *ADH* displayed positive associations with Asp., Thr, and Ser, as well as with Iso and stearic acid (FA9). Meanwhile, *LOX* exhibited positive correlations with palmitic acid (FA1), arachidonic acid (FA6), lauric acid (FA7), and heptadecanoic acid (FA8). These findings indicate that *ADH* mainly relates to amino acid metabolism while *LOX* is primarily involved in fatty acid metabolism. In addition, there is a correlation between six substances and esters, with three showing a positive correlation (FA8, *AAT*, *PLA2*) and the remaining three displaying a negative correlation (Lys, Pro, Asp). Eight substances are associated with alcohol, all of which demonstrate a positive correlation. As for aldehydes, Leu is positively correlated with eight substances, while *LOX*, *AAT*, *PLA2*, FA1, FA3, FA6, and FA7 exhibit negative correlations.

Table 2 shows a ranked list of correlations between enzymes, amino acids, fatty acids and volatile compounds. The most notable correlations were observed between enzymes and volatile compounds, with a coefficient of 0.85. Fatty acids came in second with a correlation coefficient of 0.77, followed by amino acids with 0.65. The greater *r* value noted in fatty acids than that of amino acids suggests a more robust metabolic response to fatty acid exposure compared to amino acids, following HHP processing in Hami melon juice.

**Table 2:** Correlation analysis of aroma volatile compounds and precursors in HHP treatments.

Index	*r*	*p*
Enzymes	0.85 ± 0.02	0.002
Amino acids	0.65 ± 0.03	0.006
Fatty acids	0.77 ± 0.02	0.005

## 4. Discussion

In comparison with the untreated group, the number of esters in HHP treatment decreased significantly, possibly because the pressure increased the decomposition reaction of the solvent, which nucleophilically attacked the esters and promoted their hydrolysis into various acids and alcohols (20). Similar findings have been reported in previous studies, such as the research conducted by Yi et al. (21), which indicated a significant reduction in ester levels in turbid apple juice following HHP treatment. Furthermore, HHP treatment resulted in altered levels of alcohols and aldehydes in Hami melon juice at different pressures. In contrast to other studies, Liu et al. (22) found that *6Z*-nonenal (*3E*,*6Z*)-nonadienol had no significant changes (*p* > 0.05), and (*2E*,*6Z*)-nonadienal as well as *2E*-nonenal had an obvious increase, making the cucumber odor fresher, which agreed with our results. Viljanen et al. (23) investigated the effects of 800 MPa treatment on tomatoes, revealing changes in key flavor compounds such as *3Z*-hexenal and *2Z*-pentenal. Two reasons were given for the changes in flavor caused by HHP treatment, firstly, the content of flavor compounds may have increased after pressure was applied, resulting in increased contact between enzymes and their substrates and automatic oxidation of lipids (24). Secondly, HHP treatment is likely to impact the interaction between protein and flavor compounds (21).

The changes in *LOX* observed in this study were consistent with those reported in other studies, for instance, *LOX* in tomato juice lost more activity at higher pressures (550 MPa, 12 min/600 MPa, 10 min) according to the research of Rodrigo et al. (25), the activity of *ADH* was highest after ultrahigh pressure treatment, but decreased with increasing pressure. The research conducted by Denoya et al. (26) supports our conclusion regarding the *ADH* of two varieties of freshly cut peaches. The study determined that pressure has an impact on the activity of *ADH*, with a significant decrease observed when the pressure increased from 500 MPa to 700 MPa. Enzyme activity changes aligned not only with external factors such as pressure, temperature, and duration but with enzyme properties and internal factors such as substances like ethylene and tannin in fruit. Our results, similar to previous research, were substantiated by the crucial *AAT* that transformed alcohols to esters (10, 27). Shan et al. (28) found close links between the activity of the *AAT* in various melon cultivars and their aroma compounds. They showed that this activity directly impacted the production of volatile aldehydes and alcohols.

The HHP treatment process influenced the functioning of metabolic substrates such as amino acids and fatty acids, as well as enzymes related to volatile compounds, resulting in the modification of aroma substances. According to findings by Contreras et al. (29), differences in processing methods were able to alter the release of volatile compounds by affecting the correlation between factors in amino acid and fatty acid metabolism. For instance, the compounds, such as hexyl alcohol, hexyl ester, and caproic aldehyde, along with their metabolites, originated from the actions of *LOX* on linoleic acid. Moreover, *LOX* on linolenic acid predominantly resulted in the production of *3Z*-hexenal and *2E*-hexenal. El-Hadi et al. (30) discovered that the monomer amino acids contributing to fruit aroma metabolism primarily consisted of Val, Leu, Iso, Ala, Cys, and Phe, which are consistent with the results of our study. The association of numerous amino acids with flavor substances arose primarily due to the involvement of amino acid invertase in breaking them down, which was released during protein hydrolysis, and its activities led to the production of various volatile compounds (31, 32). Zhang et al. (33) reported that some alcohols of synthetic linear esters were also derived from *LOX*, which acted on aldehydes produced by fatty acids and was reduced to alcohols with the participation of *ADH*. In Shi et al.’s study (34), the decrease in aromatic ester showed a positive correlation with the change in *ADH* activity and the levels of oleic acid and linoleic acid, and a negative correlation with the activities of lipase, phospholipase D (*PLD*), *LOX*, and the rate of membrane permeability. Wang et al. (35) found that the *LOX* in tomatoes exerted large impacts on saturated and unsaturated C6 and C9 alcohols and aldehydes, by studying the effects of high-pressure treatment on volatile ingredients and odors in tomatoes. The literature suggests that specific enzymes and substrates can influence the aroma in metabolic processes, which supports our conclusions.

## 5. Conclusion

HHP treatment had an effect on the species and contents of esters, alcohols and aldehydes in the volatile compounds of juice, mainly a substantial decrease in esters and a substantial increase in aldehydes and C6 and C9 alcohols. HHP treatment decreased the activities of four vital enzymes, of which *PLA2* and *AAT* activities were similar to *LOX*, and the activity of *ADH* was opposite to *LOX*. the activity of *ADH* was significantly increased, but decreased with the increase of pressure. The types and contents of amino acids in Hami melon juice increased, and the total amount of amino acids was also elevated, although the total amount of fatty acids decreased. By correlation analysis, there were six ester-related substances and eight alcohol-related, ketone-related and aldehyde-related substances in high pressure-treated Hami melon juice, and the correlation between fatty acids and volatile compounds was greater than the correlation between amino acids and volatile flavor compounds.

## Data availability statement

The original contributions presented in the study are included in the article/supplementary material, further inquiries can be directed to the corresponding author.

## Author contributions

LP: Methodology, Validation, Writing – original draft, Data curation, Investigation. WL: Data curation, Investigation, Writing – review & editing, Formal analysis, Validation. LJ: Conceptualization, Data curation, Writing – review & editing, Visualization. HX: Conceptualization, Data curation, Formal analysis, Writing – review & editing. LL: Data curation, Software, Visualization, Validation, Writing – review & editing. XW: Data curation, Formal analysis, Software, Writing – review & editing. ML: Software, Visualization, Writing – review & editing. BA: Investigation, Writing – review & editing, Validation. HZ: Investigation, Writing – review & editing. JC: Funding–acquisition, Methodology, Project–administration, Writing – review & editing.

## Footnotes

^1^ http://www.cytoscape.org
